# Narcolepsy type I-associated DNA methylation and gene expression changes in the human leukocyte antigen region

**DOI:** 10.1038/s41598-023-37511-4

**Published:** 2023-06-28

**Authors:** Kugui Yoshida-Tanaka, Mihoko Shimada, Yoshiko Honda, Akihiro Fujimoto, Katsushi Tokunaga, Makoto Honda, Taku Miyagawa

**Affiliations:** 1 Department of Human Genetics, Graduate School of Medicine, The University of Tokyo, Tokyo, Japan; 2 Genome Medical Science Project (Toyama), National Center for Global Health and Medicine, 1-21-1 Toyama, Shinjuku-ku, Tokyo, 162-8655 Japan; 3 Sleep Disorders Project, Department of Psychiatry and Behavioral Sciences, Tokyo Metropolitan Institute of Medical Science, Tokyo, Japan; 4 Seiwa Hospital, Institute of Neuropsychiatry, Tokyo, Japan

**Keywords:** Diseases, Neurological disorders, Genetics, Epigenetics

## Abstract

Narcolepsy type 1 (NT1) is caused by a loss of hypothalamic orexin-producing cells, and autoreactive CD4^+^ and CD8^+^ T cells have been suggested to play a role in the autoimmune mechanism. Although NT1 showed a strong association with human leukocyte antigen (*HLA*)-DQB1*06:02, the responsible antigens remain unidentified. We analyzed array-based DNA methylation and gene expression data for the *HLA* region in CD4^+^ and CD8^+^ T cells that were separated from the peripheral blood mononuclear cells of Japanese subjects (NT1, N = 42; control, N = 42). As the large number of SNPs in the *HLA* region might interfere with the affinity of the array probes, we conducted a comprehensive assessment of the reliability of each probe. The criteria were based on a previous study reporting that the presence of frequent SNPs, especially on the 3′ side of the probe, makes the probe unreliable. We confirmed that 90.3% of the probes after general filtering in the *HLA* region do not include frequent SNPs, and are thus suitable for analysis, particularly in Japanese subjects. We then performed an association analysis, and found that several CpG sites in the *HLA* class II region of the patients were significantly hypomethylated in CD4^+^ and CD8^+^ T cells. This association was not detected when the effect of HLA-DQB1*06:02 was considered, suggesting that the hypomethylation was possibly derived from HLA-DQB1*06:02. Further RNA sequencing revealed reduced expression levels of *HLA-DQB1* alleles other than HLA-DQB1*06:02 in the patients with NT1. Our results suggest the involvement of epigenetic and expressional changes in *HLA-DQB1* in the pathogenesis of NT1.

## Introduction

Narcolepsy type I (NT1) is a type of central disorder of hypersomnolence characterized by excessive daytime sleepiness and cataplexy. Onset occurs most commonly during adolescence, and its prevalence is 0.16–0.18% in the Japanese population^1^. Although the symptoms can be managed to some extent by medication, no fundamental cure has yet been established.

The pathogenesis of NT1 has been linked to the loss of orexin (also known as hypocretin) signal transduction; orexin is a neuropeptide produced exclusively in the lateral hypothalamus^2^. In human NT1, damaged hypothalamic orexin-producing cells in the postmortem brain^3^, and a significant lack of orexin A in the cerebrospinal fluid of patients^4^ have been reported. However, no causal variant has been reported in the sequences of the *prepro-orexin* and *orexin receptor* genes, except for in one rare case^3^. In fact, the concordance rate in monozygotic twins for NT1 is approximately 20–30%, and the prevalence of NT1 among first-degree relatives of patients is 1–2%, which is 10- to 40-fold higher than the risk in the general population^5^. This indicates that human NT1 is a multifactorial disease involving complex interactions between genetic and environmental factors, unlike inherited canine narcolepsy.

NT1 is known to be strongly associated with specific alleles in the human leukocyte antigen (HLA) class II region on chromosome 6. In particular, more than 90% of the patients carry the HLA-DQB1*06:02 allele^6–9^. Other than the HLA-DQB1*06:02 allele, multiple combinations of *HLA* alleles that affect the disease susceptibility have been found^6–11^. In addition to the *HLA* alleles, several immune-related factors, including *TNFSF4*, *CTSH*, *TCRA*, *P2RY11/DNMT1*, and *CCR1*/*CCR3,* have been identified by genome-wide association studies^12–16^. In different populations, immunological challenges, such as those by H1N1 AS03-adjuvanted pandemic vaccine (Pandemrix) and *Streptococcus pyogenes* infection, are considered to trigger NT1^17–25^. Moreover, the presence of orexin-specific autoreactive CD4^+^ T cells in the blood of patients with NT1^26^, and the combination of HLA-DQB1*06:02 with autoreactive CD8^+^ T cells that recognize peptides specifically expressed by orexin-producing cells have been reported^27^. In a mouse model, CD8^+^ T cells were found to attack orexin neurons, causing the loss of orexin neurons and narcoleptic symptoms^28^. These findings suggest the possibility that CD4^+^ T cells and CD8^+^ T cells trigger autoimmune reactions through their distinctive pathways in NT1.

While almost all patients with NT1 carry the HLA-DQB1*06:02 allele, it is also a relatively common allele in the general populations with a prevalence of about 12–40%. To better understand the relationship between HLA-DQB1*06:02 and NT1, it is necessary to focus on not only the nucleotide sequence, but also epigenetic regulation and gene expression. Here, we focused on DNA methylation, specifically in the *HLA* region, and investigated its association with NT1 for the following reasons. Since there is an average diagnostic delay of 10 years in NT1^29^, the gene expression profile determined at the initial visit to a sleep center might not reflect the process of the loss of orexin-producing cells. On the other hand, DNA methylation patterns that regulate gene expression are also known to serve as a cellular memory system^30^, and the patterns are expected to retain the signature of significant events on orexin neurons. Further, causal mutations in the replication foci targeting sequence domain of DNA methyltransferase 1 (DNMT1) were found in families with atypical narcolepsy and patients showed the genome-wide methylation abnormality^31^. In addition, a genome-wide association study for NT1 also reported that SNPs near *DNMT1* were genome-wide significantly associated with NT1^15^.

In this study, we performed DNA methylation analysis with blood-derived samples using array-based technology, where methylation rates were determined by the ratios of the fluorescence intensity using probes that are complementary to the sequences flanking the CpG sites. Therefore, probes designed for regions in which the sequences are highly polymorphic may not be completely complementary to the sequence. A single nucleotide polymorphism (SNP) in the probe sequence lowers the affinity, and the results can thus become inaccurate. In a previous study, the impact of SNPs in such probe sequences was investigated by examining the standard deviation of *β* values^32^. It was found that the impact is most significant when the SNP is located precisely at the CpG site of interest. Compared to areas where the SNP has no effect, the standard deviation can increase by up to two to four times^32^. Furthermore, in Illumina methylation arrays following the 450K array, there are two types of probe designs incorporated. Type I probes measure methylation levels using two types of probes, for methylated and for unmethylated, while Type II probes measure methylation levels using a single-base extension reaction. It has been reported that Type II probes are more susceptible to increased standard deviation (SD) due to the presence of SNPs. Additionally, the SD is more likely to increase with higher minor allele frequencies of the SNPs present. However, it has been observed that a SNP located more than 10 nucleotides away from the CpG site of interest has little effect on the results^32^.

Although the *HLA* region plays an essential role in the pathogenesis of NT1, the large number of SNPs and the large differences between populations in the allele frequencies of SNPs in the *HLA* region make it difficult to analyze the data from microarrays when compared to other genomic regions. Our previous methylation study of NT1 in blood samples using Infinium 27K methylation array thus removed *HLA* region from the analyses^33^. Here we suggested a method of rigorous analysis of DNA methylation in the *HLA* region, and conducted a more precise determination of DNA methylation rates of CD4^+^ and CD8^+^ T cells using Infinium methylation EPIC array (> 860K probes). To evaluate the SNP-dependent reliability of each probe in the *HLA* region, we first confirmed the presence of SNPs in the probes for the Japanese population, then performed an association analysis to search for methylation patterns related to NT1. In addition, since the methylation rate of the *DQB1* region was found to be associated with NT1 in both CD4^+^ T cells and CD8^+^ T cells, we also determined the gene expression levels of *HLA*-*DQB1* to examine the functional relevance of the observed change in the methylation status*.*

## Material and methods

### Participants and samples for the DNA methylation analysis

Peripheral blood-derived samples from 42 Japanese patients with NT1 and 42 healthy volunteer controls were collected at Seiwa Hospital affiliated with the Institute of Neuropsychiatry (Shinjuku-ku, Tokyo, Japan). The patients were diagnosed by physician sleep specialists based on the International Classification of Sleep Disorders, 3rd edition^34^. All patients were HLA-DQB1*06:02-positive and free of medication affecting sleep at the time of blood collection. The demographic characteristics of the samples are presented in Supplementary Table 1. We separated the CD4^+^ T cells and CD8^+^ T cells from the blood samples using the Dynabeads CD4 Positive Isolation Kit and Dynabeads CD8 Positive Isolation Kit (Invitrogen, CA), respectively, according to the manufacturer’s protocols. DNA was extracted from the CD4^+^ or CD8^+^ T cells using a QIAamp DNA Mini Kit (QIAGEN, Germany). This study was approved by the ethics committees of the Institute of Neuropsychiatry, Tokyo Metropolitan Institute of Medical Science, and the University of Tokyo. All participants provided written informed consent for participation in this study and all methods were performed in accordance with the relevant guidelines and regulations.

### Determination of the DNA methylation rates and data processing

The DNA methylation rates of the samples were determined using array-based technology (Infinium Methylation EPIC BeadChip, Illumina, Inc., San Diego, CA). In the array-based method, unmethylated cytosine is converted to uracil by sodium bisulfite. Then, the DNA samples are fragmented and hybridized to locus-specific probes, followed by allele-specific extension. A high-precision scanner (iScan System, Illumina, Inc.) was used to detect the signal intensities of the biotin and 2,4-dinitrophenol labeled on dideoxynucleotides incorporated in the single-base extension. The output data of the signal intensities were transformed to IDAT files by GenomeStudio Software (Illumina, Inc.), imported with the R package ChAMP, and used to calculate the ratio of the intensity from methylated cytosine probes to the total signal intensity (β values) as the methylation rate^35^. Detection P values were determined by an unconventional method that has improved sensitivity to aberrant values^36^. The probes that failed to determine the methylation rate with a detection P value of ≥ 0.01 in more than 10% of the samples were omitted. Furthermore, we filtered out the probes that have been reported to be unreliable, such as probes with low mapping quality, and probes with copy number variations and SNPs^32,36^. Probe design bias between type I and type II probes was normalized with BMIQ^37^, and batch effects and distributional variability were standardized by Combat^38^ and quantile normalization, respectively. We analyzed the methylation rate data of the *HLA* region in both CD4^+^ and CD8^+^ T cells.

### RNA samples for expression analysis

RNA was extracted from the whole blood of the same participants. The whole blood was collected into PAXgene Blood RNA tubes (Becton Dickinson, UK), and total RNA was isolated using the PAXgene Blood RNA Kit (QIAGEN) according to the manufacturer’s protocol. The RNA integrity number (RIN) and concentration were assessed using an Agilent 2100 Bioanalyzer (Agilent Technologies, CA). RNA samples with an RIN of > 7 were used for RNA sequencing.

### Examination of the reliability of probes

We focused on the *HLA* region and its flanking region (Chr6: 26,000,000–35,000,000, GRCh37/hg19), and included 12,181 methylation array probes in the region for the analyses.

To assess the validity of these probes with consideration of the high polymorphism of the *HLA* region, we first examined the SNPs that are found frequently in the Japanese population in the complementary sequences of the probes. We used the 4.7KJPN Allele Frequency Panel (tommo-4.7kjpn-20190826-MAF_snvall-autosome.vcf.gz (SNV; Autosome)) from the Tohoku Medical Megabank (https://jmorp.megabank.tohoku.ac.jp) as a reliable database of the SNPs in the Japanese population with a large sample size (n = 4773). We also obtained the chromosomal positions of the sequences to which the probes bind from the annotation file (Infinium MethylationEPIC v1.0 B5 Manifest File) provided by Illumina Inc. We then examined the presence of SNPs with a minor allele frequency (MAF) larger than 0.05 and 0.01 in the target site of each probe.

The effect of the presence of a SNP on annealing, which lowers the reliability of the results, is largest when the SNP is located on the 3′ end of the probe. The effect decreases exponentially with increased distance from the 3′ end, and becomes negligible when the SNP is located more than 10 nt away from 3′ end^32^. Therefore, we considered the region within 10 nt from the 3′ end of a probe to be influential, and we searched for probes with SNPs that are frequent in the Japanese population and are located in the complementary sequence of the influential region. We classified the influential region into three groups: (1) “ONSITE” for the SNPs that have the strongest effect on the affinity; (2) “5nt” for the SNPs that have a moderate effect on the affinity; and (3) “10nt” for the SNPs that have a small effect on the affinity.

**Figure 1:**
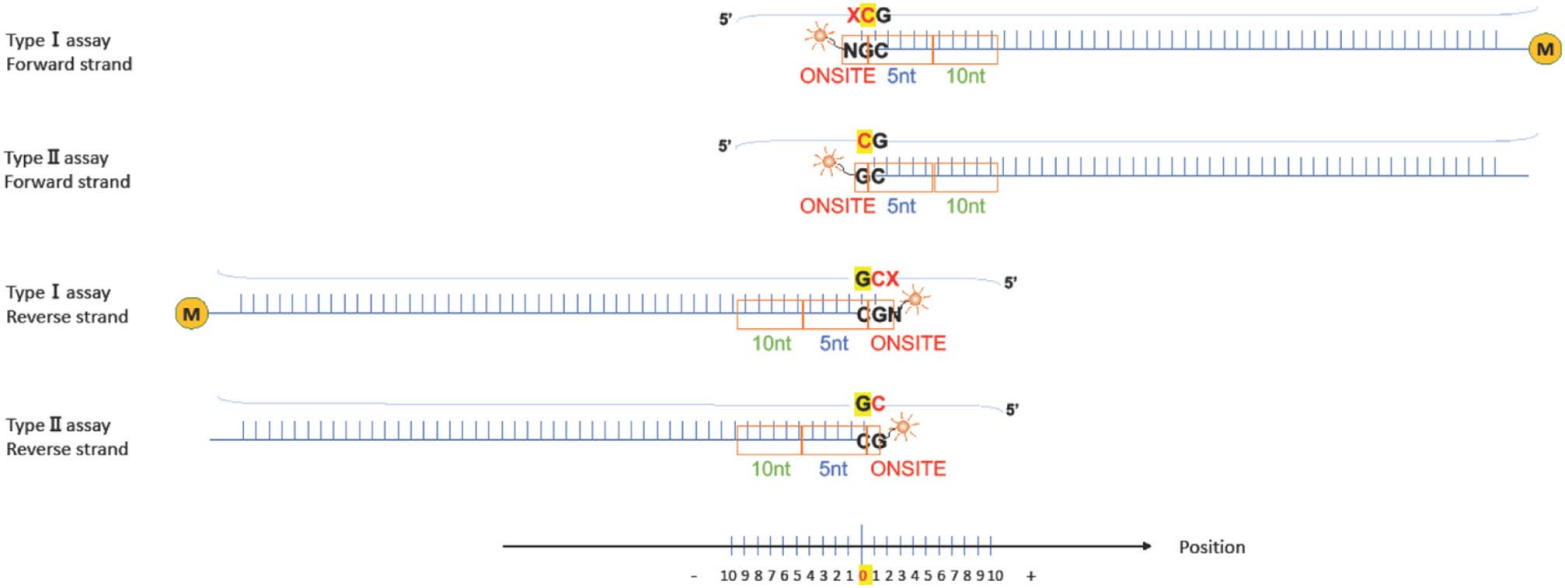
Scheme for detecting unreliable probes. The numbers are the positions relative to the probe-binding positions described in the annotation file (marked in yellow). We examined SNPs that were located in the possibly influential region (indicated by orange boxes) that affects probe reliability. The SNPs were classified according to their location: (1) “ONSITE” SNPs are located in the targeted CpG site or the flanking base where the fluorescent probe binds (Type I); (2) “5nt” SNPs are located within 1 to 5 nt from the target site; and (3) “10nt” SNPs are located within 6 to 10 nt from the target site. The examined positions were as follows. In the Type I assay—Forward strand: (1) − 1 and 0, (2) 1 to 5, and (3) 6 to 10; in the Type II assay—Forward strand: (1) 0, (2) 1 to 5, and (3) 6 to 10; in the Type I assay—Reverse strand: (1) 1 and 2, (2) − 4 to 0, and (3) − 9 to − 5; and in the Type II assay—Reverse strand: (1) 1, (2) − 4 to 0, and (3) − 9 to − 5. A probe designed for the methylated locus (M bead type) is shown as an example for the Type I assay.

In the annotation file, the binding position of the probe indicates the cytosine of the target CpG sequence in the forward strand, and the guanine next to the cytosine in the reverse strand. Therefore, we created a Python program that can handle four cases: forward or reverse orientation of the strand, and Type I or Type II assays (Fig. 1).

### DNA methylation association analysis

The DNA methylation rates in the *HLA* regions of CD4^+^ T cells and CD8^+^ T cells of the patients and controls were first compared by a *t*-test. Next, considering the significant difference in the positivity rate for the HLA-DQB1*06:02 allele between the patients (42/42) and the controls (8/42), the effect of the allele was corrected by regression analysis. As the analyses comprised multiple testing for 12,181 probes, the significance level was set at *α* = 4.06 × 10^−6^ (0.05/12,181) with Bonferroni correction. We located the *HLA* class I region as the region from the 5′ end of *HLA-F* to the 5′ end of *HLA-B*, and the *HLA* class II region as the region from the 5′ end of *HLA-DRA* to the 3′ end of *HLA-DPB2* in the human assembly GRCh37/hg19 (Chr6: 29,691,241–31,323,369, and Chr6: 32,407,619–33,086,926, respectively). R (version 4.0.3) was used for the analyses.

### Expression analysis

We performed RNA sequencing to analyze the expression profiles of the *HLA* genes in the 42 patients and 42 controls. Sequencing libraries were prepared with TruSeq Stranded Total RNA Ribo Zero (Illumina Inc.). Paired-end RNA sequencing was conducted on NovaSeq 6000 (Illumina Inc.). Raw FASTQ data were processed with CLC Genomics Workbench 21 (Qiagen). The reads were trimmed using a quality score of 0.05 and a minimum length of 15 nt. The trimmed reads were mapped to the reference sequence with the following parameters: mismatch cost, 2; insertion cost, 3; deletion cost, 3; length fraction, 0.8; similarity fraction, 0.8; and maximum number of hits for a read, 10. The read count data were normalized by the trimmed mean of M values in the package edgeR^39–41^, and the expression status of the patients and controls was compared by the likelihood ratio test. The design matrix included the age, sex, and body mass index as covariates. We also used arcasHLA^42^ to type the *HLA* allele to compare the quantity of the *DQB1*, *DQA1*, *DRB1*, *DRA*, *DPB1* and *DPA1* genes by allele. The read count data were normalized by allele lengths, and converted into transcript abundances. The transcript abundances were standardized so that the total read number was 1 million, and they were used for the comparison of the expression levels per allele, and between the patients and controls.

## Results

### Reliability of probes in the *HLA* region

**Figure 2:**
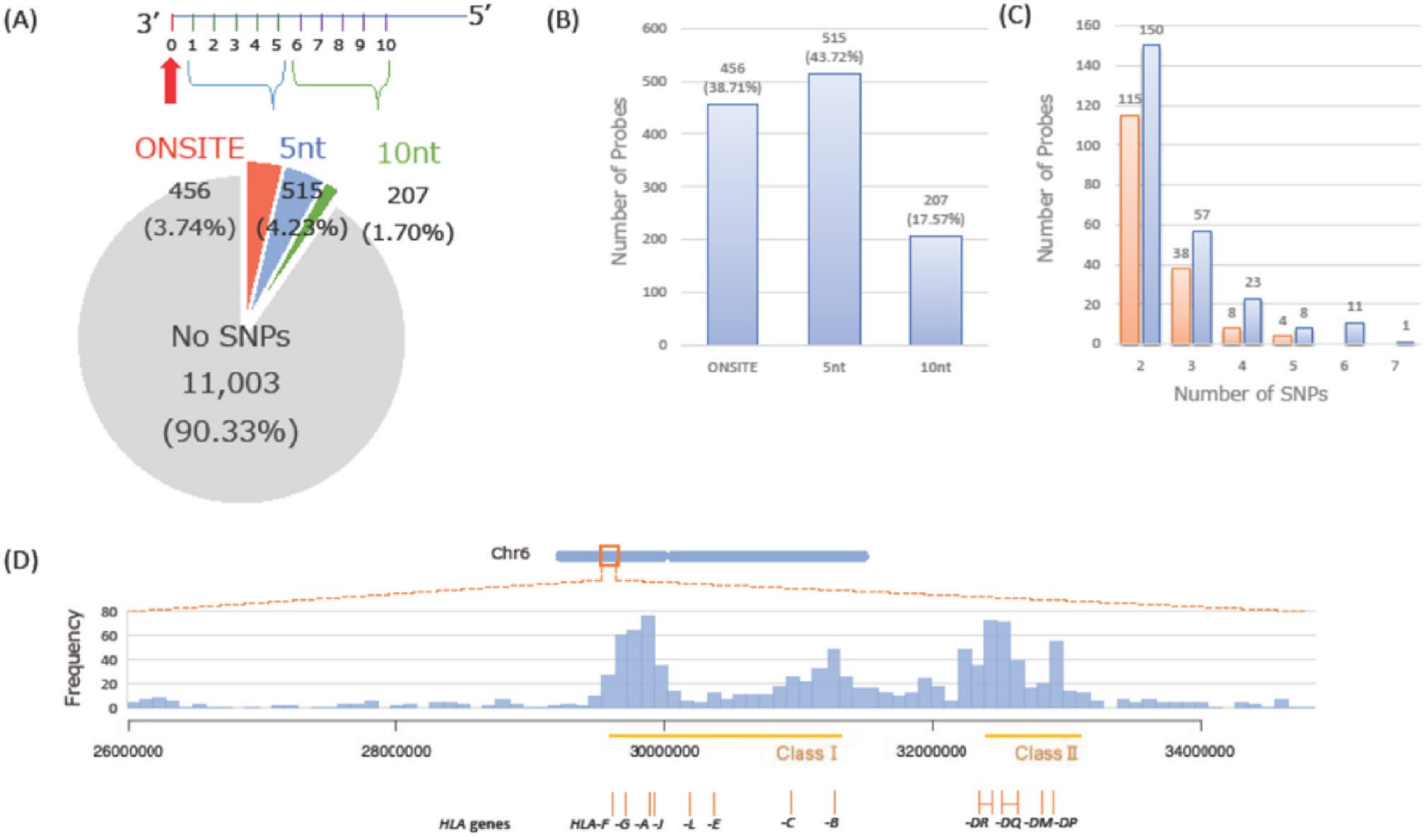
Examination of the reliability of probes (MAF ≥ 0.01). The examination results of the SNPs with a MAF ≥ 0.01 as the baseline. (**A**) Number of SNPs classified according to the distance from the 3′ end of the probe. More than 90% of the probes after general filtering did not have any SNP in the influential region. (**B**) Number of probes that had one or more SNPs. Many of the SNPs were located in “ONSITE” or “5nt”. (**C**) Number of probes that had multiple SNPs within “ONSITE”, “5nt”, and “10nt” (blue) and “ONSITE” and “5nt” (red). These probes were theoretically estimated to have low reliability due to their high polymorphism. (**D**) Histogram of the number of probes that had one or more SNPs within “ONSITE”, “5nt”, and “10nt” in the *HLA* region. Most of them targeted CpG sites in the Class I and Class II region.

We assessed whether 12,181 probes in the *HLA* region on the Infinium methylation EPIC Beadchip have polymorphic nucleotides inside their sequences in the Japanese population. With the standard of a MAF ≥ 0.05 in the Japanese population, we found 370 probes that had a SNP on the 3′ end, 397 probes within 1 to 5 nt from the 3′ end, and 166 probes within 6 to 10 nt from the 3′ end (Supplementary Fig. 1). With the standard of a MAF ≥ 0.01 in the Japanese population, we found 456 probes that had a SNP on the 3′ end, 515 probes within 1 to 5 nt from the 3′ end, and 207 probes within 6 to 10 nt from the 3′ end (Fig. 2A,B).

We adopted the more conservative standard, and focused on the results from SNPs with a MAF ≥ 0.01. Notably, 21.22% of the probes (250/1178) contained multiple SNPs within 10 nt from the 3′ end. More interestingly, 14.01% of the probes (165/1178) contained multiple SNPs within 5 nt from the 3′ end (Fig. 2C). The majority of the probes that had SNPs within 10 nt from the 3′ end targeted the CpG sites in the *HLA-A* region (29,900,000–30,000,000), the *HLA-DRB1* region (32,500,000–32,600,000), and the *HLA-DQB1* region (32,600,000–32,700,000; Fig. 2D). However, 96.26% of the probes (2754/2861) in the *HLA* class I region, and 92.58% of the probes (824/890) in the *HLA* class II region did not contain any influential SNPs, confirming the reliability of such probes.

We considered these 1178 probes to be unreliable, and distinguished them in the following analyses to exclude them from the interpretation of the results.

### Methylation analysis in the *HLA* region

**Figure 3:**
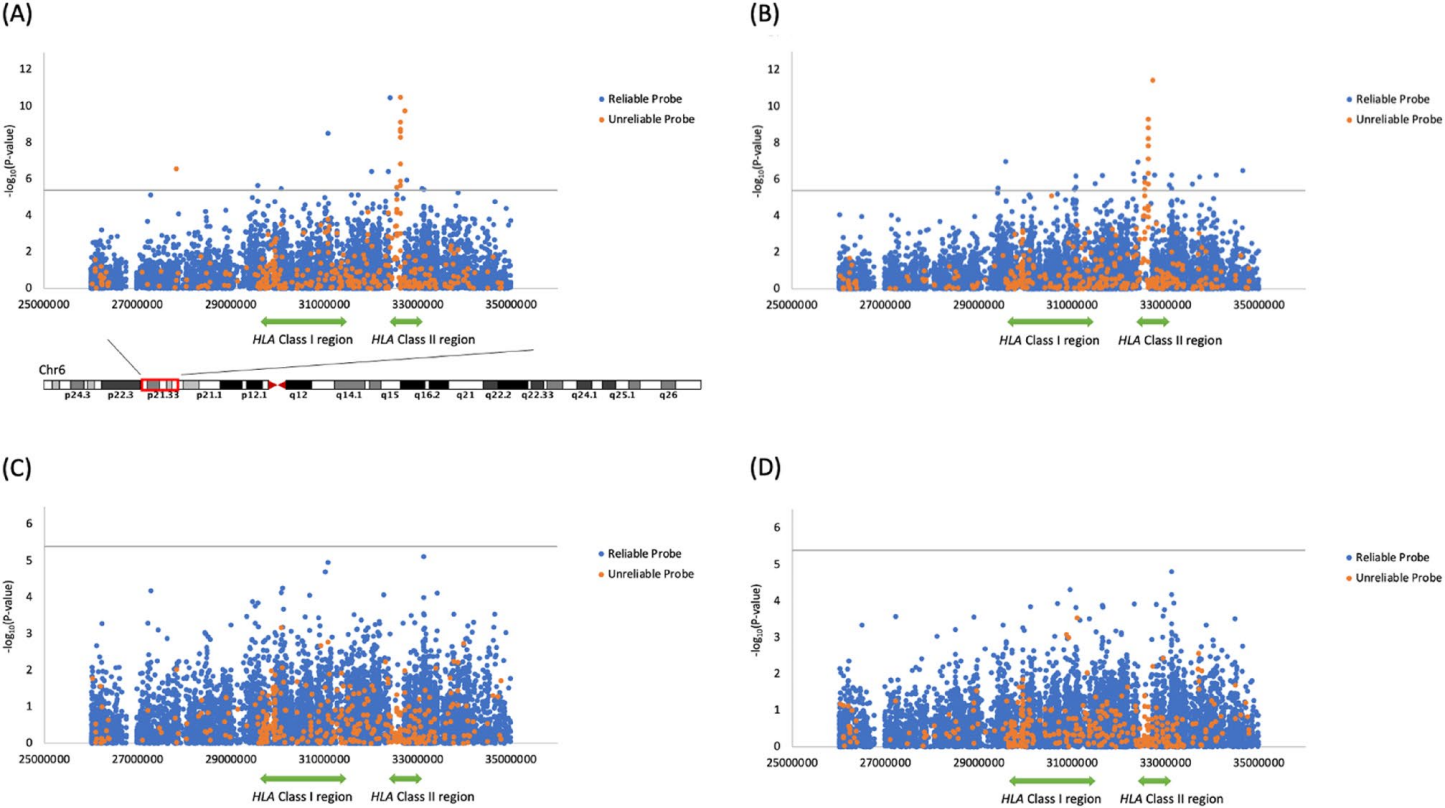
Manhattan plots of P-values for association analyses. Reliable probes that do not have SNPs in the sequence and yield trustworthy results are indicated in blue. Probes that contain SNPs and have low result reliability are indicated in orange. The significance level is represented by the gray line. In the analysis without adjusting for the presence of HLA-DQB1*06:02, multiple probes exceeding the significance level were observed in both cell types (**A**,**B**). Many of these were found to be unreliable probes. In the analysis adjusted for the presence of HLA-DQB1*06:02, no probes indicating an association with NT1 exceeded the significance level (**C**,**D**). (**A**) Result of *t*-test determining the association of NT1 in CD4^+^ T cells. The influence of HLA-DQB1*06:02 has not been adjusted for. (**B**) Result of *t*-test determining the association of NT1 in CD8^+^ T cells. The influence of HLA-DQB1*06:02 has not been adjusted for. (**C**) Result of regression analysis determining the association of NT1 in CD4^+^ T cells, adjusting for the influence of HLA-DQB1*06:02. (**D**) Result of regression analysis determining the association of NT1 in CD8^+^ T cells, adjusting for the influence of HLA-DQB1*06:02.

Regarding the differences in the DNA methylation rates between the patients and controls, a peak *P* value was seen for the probes from the *HLA* class II region in both CD4^+^ T cells and CD8^+^ T cells (Fig. 3A,B).

There were 1893 probes for CD4^+^ T cells and 1923 probes for CD8^+^ T cells that showed a *P* value < 0.05. Among these probes, 905 probes were in common between CD4^+^ T cells and CD8^+^ T cells. After removing the unreliable probes with the most conservative baselines (SNPs with a MAF > 0.01 and within 10 nt from the 3′ end), 1809 probes for CD4^+^ T cells and 1843 probes for CD8^+^ T cells showed a *P* value < 0.05 (844 probes were in common). Among these nominally significant probes, 77.9% (1410/1809) of the probes for CD4^+^ T cells and 82.5% (1520/1843) of the probes for CD8^+^ T cells showed lower methylation rates in the patients than in the controls. Nine probes for CD4^+^ T cells and 19 probes for CD8^+^ T cells remained significant after Bonferroni correction (3 probes were in common). The probe with the lowest *P* value in CD4^+^ T cells was cg00383136 (*P* = 3.5 × 10^−11^), which was also significantly associated in CD8^+^ T cells (*P* = 1.12 × 10^−7^), and that in CD8^+^ T cells was cg00667298 (*P* = 1.08 × 10^−7^), which was also significantly associated in CD4^+^ T cells (*P* = 2.3 × 10^−6^). The target site of cg00383136 was in the 5′ untranslated region of *HLA-DRA*, and the mean methylation rate of the patients was 4.0% lower than that of the controls. On the other hand, the target site of cg00667298 was on the 16th intron of gamma-aminobutyric acid B receptor, 1 (*GABBR1*), and the mean methylation rate of the patients was 4.9% lower than that of the controls (Table 1, Supplementary Tables 2, 3).

**Table 1:** Reliable probes with significantly different β values before the HLA-DQB1*06:02 adjustment.

Position	Probe	Mean β value			P value	Gene	CpG position
		Control	Case	Difference			
CD4^+^ T cells							
32410247	cg00383136	0.8049	0.7651	− 0.0398	3.5E−11	*HLA-DRA*	Body
31079895	cg17663033	0.9252	0.8947	− 0.0305	3.06E−09	*C6orf15*	Body
32016360	cg05298224	0.4677	0.4347	− 0.0330	3.79E−07	*TNXB*	Body
32375086	cg18286996	0.8251	0.7926	− 0.0326	3.81E−07	*BTNL2*	TSS200*
32762284	cg00699601	0.3448	0.5265	0.1817	1.15E−06		
29576329	cg00667298	0.8283	0.795	− 0.0328	2.3E−06	*GABBR1*	Body
30080295	cg09816018	0.7215	0.6830	− 0.0384	3.3E−06	*TRIM31*	Body
33095664	cg02388468	0.8488	0.8265	− 0.0223	3.44E−06	*HLA-DPB2*	Body
33135763	cg09344631	0.8829	0.8645	− 0.0184	3.7E−06	*COL11A2*	Body
CD8^+^ T cells							
29576329	cg00667298	0.7850	0.7361	− 0.0489	1.08E−07	*GABBR1*	Body
32410247	cg00383136	0.8319	0.8044	− 0.0276	1.12E−07	*HLA-DRA*	Body
34649209	cg21858187	0.7429	0.7130	− 0.0299	3.22E−07	*C6orf106*	Body
32317028	cg16905004	0.8280	0.7848	− 0.0432	4.77E−07	*C6orf10*	Body
32762284	cg00699601	0.3551	0.5454	0.1903	5.68E−07		
34081581	cg07545195	0.6437	0.6043	− 0.0394	5.75E−07	*GRM4*	Body
33132318	cg10537192	0.6060	0.5757	− 0.0304	5.88E−07	*COL11A2*	Body
31646061	cg03337084	0.6176	0.5936	− 0.0240	6.11E−07	*LY6G5C*	Body
31081323	cg13060500	0.8015	0.7617	− 0.0399	6.41E−07	*PSORS1C1*;*C6orf15*	TSS1500;TSS1500*
33724626	cg01038670	0.6685	0.6270	− 0.0415	7.32E−07		
32551749	cg11404906	0.4032	0.1466	− 0.2566	8.21E−07	*HLA-DRB1*	Body
32335123	cg19886087	0.8252	0.7987	− 0.0265	1.24E−06	*C6orf10*	Body
31496949	cg12259379	0.6504	0.6061	− 0.0444	1.7E−06	*MCCD1*	1st Exon
33576885	cg11428942	0.8204	0.7953	− 0.0252	1.79E−06		
33080054	cg24310786	0.5768	0.5452	− 0.0316	2.02E−06	*HLA-DPB2*	TSS1500*
31080529	cg16150435	0.8927	0.8728	− 0.0199	2.62E−06	*C6orf15*	TSS200*
29416095	cg04745064	0.6952	0.6288	− 0.0664	3.02E−06		
33132241	cg12945707	0.7264	0.6996	− 0.0268	3.18E−06	*COL11A2*	Body
31050373	cg00020172	0.8536	0.8109	− 0.0427	3.53E−06		

*TSS200 and TSS1500 refers to 0–200 bp or 200–1500 bp upstream of the transcriptional start site, respectively.

We performed a regression analysis to adjust for the effect of the HLA-DQB1*06:02 allele, because there was a significant difference in the frequency of HLA-DQB1*06:02 between the patients and controls. As a result, the peak *P* value observed before adjustment was not confirmed, and no significant association was observed for either CD4^+^ T cells or CD8^+^ T cells after Bonferroni correction (Fig. 3C,D). Most of the probes that were significant on the *t*-test after Bonferroni correction were nominally significant after controlling for the effect of HLA-DQB1*06:02. However, cg00699601 in both CD4^+^ T cells and CD8^+^ T cells, and cg11404906 in CD8^+^ T cells showed significant differences on the *t*-test, and exhibited *P* values > 0.05 on the regression analysis. In particular, the associations of HLA-DQB1*06:02, which was used as a covariate in the regression analysis, were significant in the cg00699601 analyses in both CD4^+^ T cells and CD8^+^ T cells, indicating that the methylation levels of cg00699601 were strongly associated with the presence or absence of HLA-DQB1*06:02 (Table 2).

**Table 2:** Result of the regression analysis adjusting the effect of the HLA-DQB1*06:02.

Position	Probe	NT1		DQB1*06:02 (covariate)
		Regression coefficient	P value	P value
CD4^+^ T cells				
31079895	cg17663033	− 0.0380	1.12E−05	0.269
30080295	cg09816018	− 0.0563	7.47E−05	0.112
29576329	cg00667298	− 0.0453	0.0001	0.1837
33135763	cg09344631	− 0.0248	0.0003	0.2387
32375086	cg18286996	− 0.0280	0.0091	0.5966
33095664	cg02388468	− 0.0186	0.0219	0.5711
32410247	cg00383136	− 0.0201	0.026	0.0087
32016360	cg05298224	− 0.0232	0.0309	0.2609
32762284	cg00699601	− 0.0964	0.0515	9.88E−10
CD8^+^ T cells				
33132241	cg12945707	− 0.0395	6.68E−05	0.106
32335123	cg19886087	− 0.0360	0.0001	0.1981
31646061	cg03337084	− 0.0315	0.0001	0.2508
31080529	cg16150435	− 0.0276	0.0002	0.1845
33132318	cg10537192	− 0.0350	0.0007	0.5752
34649209	cg21858187	− 0.0310	0.0017	0.8907
29576329	cg00667298	− 0.0479	0.0019	0.9304
31050373	cg00020172	− 0.0468	0.0029	0.7437
33724626	cg01038670	− 0.0410	0.0038	0.9617
34081581	cg07545195	− 0.0384	0.0039	0.9262
29416095	cg04745064	− 0.0682	0.0049	0.9276
33080054	cg24310786	− 0.0313	0.0055	0.9802
31081323	cg13060500	− 0.0371	0.0059	0.8005
32410247	cg00383136	− 0.0236	0.0063	0.5703
31496949	cg12259379	− 0.0425	0.0068	0.8837
33576885	cg11428942	− 0.0231	0.0094	0.7786
32317028	cg16905004	− 0.0370	0.01	0.592
32762284	cg00699601	− 0.0887	0.0784	1.64E−09
32551749	cg11404906	− 0.0054	0.9434	0.0001

The change in P values of the probes of the Table 1 that showed significant differences in the comparison without the corrections when the effect of DQB1 allele was corrected.

The characteristics of the probes showing nominal associations were as follows. There were 1293 probes for CD4^+^ T cells and 974 probes for CD8^+^ T cells that showed a *P* value < 0.05 (276 probes were in common). After removal of the unreliable probes, 1244 probes for CD4^+^ T cells and 952 probes for CD8^+^ T cells showed a *P* value < 0.05 (266 probes were in common). Of these reliable probes with a *P* value < 0.05, 73.1% (909/1244) of the probes for CD4^+^ T cells and 75.0% (714/952) of the probes for CD8^+^ T cells showed hypomethylation in the patients. The probe with the lowest *P* value in CD4^+^ T cells was cg12945707 (*P* = 7.71 × 10^−6^), which was also nominally significant in CD8^+^ T cells (*P* = 6.68 × 10^−5^), and that in CD8^+^ T cells was cg13478289 (*P* = 1.54 × 10^−5^), which was also nominally significant in CD4^+^ T cells (*P* = 7.58 × 10^−4^); the target site of each probe was on the 65th and 64th exon, respectively, of *Homo sapiens* collagen, type XI, alpha 2 (*COL11A2*), and the methylation rates were lower in the patients than in the controls (Table 3, Supplementary Tables 2, 3).

**Table 3:** Reliable probes with lowest P values with the HLA-DQB1*06:02 adjustment.

Position	Probe	Regression coefficient	P value	Gene	CpG position
CD4^+^ T cells					
33132241	cg12945707	− 0.041	7.71E−06	*COL11A2*	Body
31079895	cg17663033	− 0.0380	1.12E−05	*C6orf15*	Body
31026105	cg08448622	− 0.0576	2.01E−05	*HCG22*	Body
30104982	cg05681072	− 0.0276	5.55E− 05	*TRIM40*	1st Exon
27291363	cg07968979	0.0357	6.57E−05	*FKSG83*	TSS1500
30080295	cg09816018	− 0.0563	7.47E−05	*TRIM31*	Body
33422266	cg16463733	− 0.0157	7.55E−05	*ZBTB9*	TSS200
32272788	cg10453756	0.0534	8.65E−05	*C6orf10*	Body
30690501	cg19748937	− 0.0112	8.88E−05	*TUBB*	Body
33132318	cg10537192	− 0.0380	0.0001	*COL11A2*	Body
CD8^+^ T cells					
33132650	cg13478289	− 0.0397	1.54E−05	*COL11A2*	Body
30956221	cg07491399	0.0778	4.79E−05	*MUC21*	3′UTR
33132241	cg12945707	− 0.0395	6.68E−05	*COL11A2*	Body
33179767	cg12565534	0.0082	0.0001	*RING1*	Body
30684931	cg10277195	− 0.0096	0.0001	*MDC1*	5′UTR
32335123	cg19886087	− 0.0360	0.0001	*C6orf10*	Body
32806921	cg08557029	− 0.0375	0.0001	*TAP2*	TSS1500
31646061	cg03337084	− 0.0315	0.0001	*LY6G5C*	Body
30112776	cg08116408	− 0.0538	0.0001	*TRIM40*	Body
31656279	cg20149270	− 0.0134	0.0001	*ABHD16A*	Body

### Expression analysis of *HLA* genes

Many of the probes that showed a significant association with NT1 before the HLA-DQB1*06:02 adjustment lost their significance after the adjustment. The methylation levels of the probes are correlated with the presence of HLA-DQB1*06:02, and changes in the methylation levels suggest changes in the HLA-DQB1*06:02 expression levels. In addition, several probes around the *HLA* genes remained nominally significant after controlling for the effects of HLA-DQB1*06:02. The methylation sites on such probes might also modify the expression levels of *HLA* genes, and contribute to the development of NT1. Therefore, we performed RNA sequencing to investigate the expression levels of *HLA* genes in the whole blood of the same patients and controls as the methylation study. The total *HLA-DQB1* gene expression level was significantly higher in the patients than in the controls (*P* = 0.018, log fold-change = 0.422). In addition, the *HLA-DPB1* gene expression level was lower in the patients than in the controls (*P* = 0.029, log fold-change = − 0.218). The *HLA-DRB1* gene is known to exhibit strong linkage disequilibrium with the *HLA-DQB1* gene, and particularly HLA-DRB1*15:01-DQB1*06:02, in the Japanese population, which was expressed at levels that were not significantly different between the patients and controls (Supplementary Table 4).

**Figure 4:**
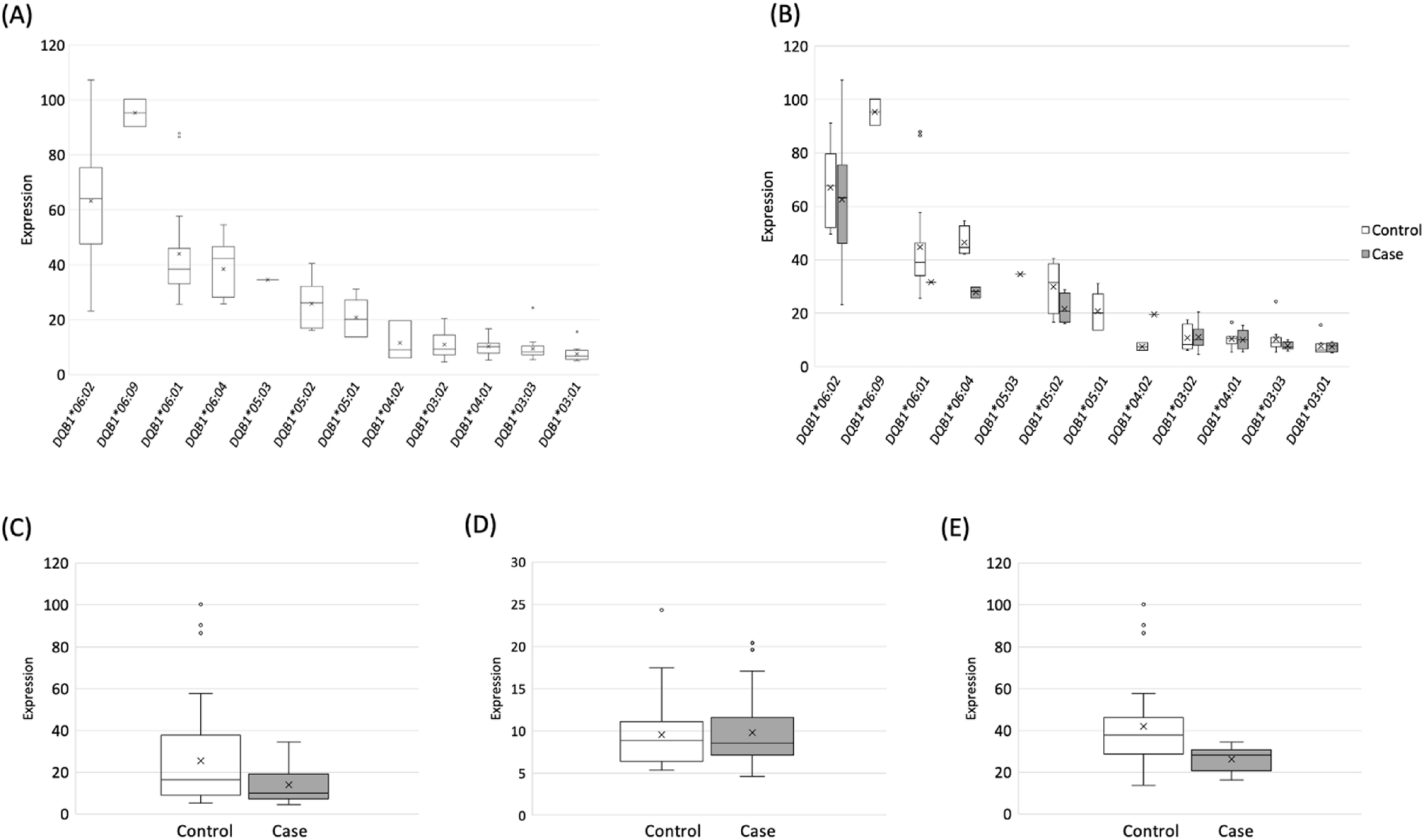
The expression levels of the *HLA-DQB1* alleles. The expression levels refer to the transcript abundances calculated from the read counts normalized by allele lengths with arcasHLA, and the total read number standardized to 1 million. (**A**) The distribution of the normalized expression levels of *HLA-DQB1* alleles were determined. The expression level of each allele was compared to that of HLA-DQB1*06:02 by the Wilcoxon rank sum test. All alleles, except for HLA-DQB1*05:03 and HLA-DQB1*06:09, showed a significant difference (*P* value < 0.05). (**B**) The expression levels of the *HLA-DQB1* alleles in the patients and controls. (**C**) The expression levels of *HLA-DQB1* alleles other than HLA-DQB1*06:02 in the patients and controls. The means of the two groups were compared by a *t*-test (*P* = 2.94E−04). (**D**) The expression levels of the HLA-DQB1*03 and *04 group in the patients and controls. The means of the two groups were compared by a *t*-test (*P* = 0.820). (**E**) The expression levels of the HLA-DQB1*05 and *06 group in the patients and controls. The means of the two groups were compared by a *t*-test (*P* = 7.53E−04).

Considering the importance of alleles in NT1, and that *HLA-DQB1* expression is subject to change according to the *DQB1* allele, we determined the *HLA* subtypes of the participants based on the RNA sequencing data, and estimated the expression level of each *HLA* allele. All of the *HLA-DQB1* alleles, except for HLA-DQB1*06:09, had significantly lower expression levels than the HLA-DQB1*06:02 allele (*P* < 0.05). HLA-DQB1*03/04 molecules are characterized by the amino-acid sequence QLELRTT at positions 84–90, while HLA-DQB1*05/06 molecules have the amino-acid sequence EV-RGI at the positions. HLA-DQB1*03/04 and HLA-DQB1*05/06 are referred to as serologically, functionally and evolutionarily distinct sublineages^43,44^. The HLA-DQB1*03 and HLA-DQB1*04 alleles had relatively lower expression levels than the HLA-DQB1*05 and HLA-DQB1*06 alleles (Fig. 4A). We then compared the expression levels of the *HLA-DQB1* alleles between the patients and controls. HLA-DQB1*06:02 was highly expressed in both groups, with no difference between the groups (Fig. 4B). The expression levels of *HLA-DQB1* alleles other than HLA-DQB1*06:02 were significantly lower in the patients than in the controls (*P* = 2.94 × 10^−4^; Fig. 4C). We also performed a stratified analysis of HLA-DQB1*03 and *04* (lower expression group), and HLA-DQB1*05 and *06* (higher expression group). No significant differences were observed in DQB1*03 and *04* between the patients and controls (*P* = 0.820; Fig. 4D). Meanwhile, we found that the expression levels of DQB1*05 and *06* were significantly lower in the patients than in the controls (*P* = 7.53 × 10^−4^; Fig. 4E). The expression levels of the *HLA-DPB1* and *HLA-DRB1* alleles were also compared between the patients and controls, but no significant differences were detected (Supplementary Figs. 2, 3).

## Discussion

In this study, we analyzed the DNA methylation and gene expression levels of *HLA* genes. We found that some CpG sites in the *HLA* class II region were hypomethylated, and that the expression levels of *DQB1* alleles other than DQB1*06:02 were low in patients with NT1. Genes in the *HLA* region are known to play an important role in the pathogenesis of NT1, as is the presence of autoreactive CD4^+^ T cells and CD8^+^ T cells. However, DNA methylation in the *HLA* region of T cells has never been examined in patients as the analysis is difficult to perform due to the high polymorphism of the region and the low reliability of the methylation rate data from both bisulfite sequencing and DNA methylation array methods, which are the main methods for performing comprehensive genome-wide DNA methylation analyses. We identified a set of unreliable methylation array probes by examining the precise locations of Japanese-specific SNPs and targeted CpG sites in methylation arrays. This enabled us to further analyze the differences in DNA methylation between the patients and controls.

A previously reported array-based methylation analysis revealed that the presence of a SNP, particularly those with a MAF > 0.05, can strongly affect the affinity of probes, yet the effect decreases with increasing distance from the 3′ end, and the effect is negligible for SNPs more than 5 nt away from the 3′ end^32^. As the location of SNPs is critical for the reliability of the probes, we classified the SNPs into three groups according to their locations: “ONSITE”, “5nt”, and “10nt”. Furthermore, we assessed the SNPs with a MAF ≥ 0.05 and those with a MAF ≥ 0.01, as the MAF also critically affects the reliability. Referring to a previous study^32^, sufficient reliability may have been attained by removing only the probes that included one or more SNPs with a MAF ≥ 0.05 in the Japanese population that were located either “ONSITE” or “5nt”. However, in this study, we adopted conservative standards, and considered the probes that contained any SNPs with a MAF ≥ 0.01 in the Japanese population that were located either “ONSITE”, “5nt”, or “10nt” to be unreliable for the analyses.

When we assessed the target sites of the probes that contained the possibly unreliable SNPs, we found that they were densely located around the *HLA-A* gene in the class I region and the *HLA-DR* gene and *HLA-DQ* gene in the class II region. This raised concerns on the reliability of the probes in these highly polymorphic regions. Indeed, in the class II region, the case–control comparisons which included unreliable probes showed that 76.9% of probes with significant associations after Bonferroni correction for CD4^+^ T cells were unreliable probes and 71.4% for CD8^+^ T cells. This highlights the impact of unreliable probes on the analysis results and underscores the importance of excluding such probes from the analysis. We also showed that some probes had more than two SNPs in influential positions (Fig. 2C). The significant proximity of these SNPs suggests the possibility that they are in linkage disequilibrium. In other words, the subjects with minor alleles for SNPs in the binding sequence of probes are likely to possess minor alleles for all SNPs in the probe due to linkage disequilibrium. These probes need careful handling. Furthermore, the number of samples from each population in the 1000 Genomes Project (around 100 subjects) is not large enough for detecting unreliable probes. Therefore, it is important to determine unreliable probes based on sufficient population-specific genomic information, as was done in our study. Yet, there were some probes in these highly polymorphic regions that were considered to be reliable, such as cg00383136, which has a target CpG site in the *HLA-DRA* gene. In fact, this probe showed a significantly strong association (*P* = 3.50 × 10^−11^) with NT1. This suggests the necessity of thoroughly examining in detail SNPs for the population of interest to determine which probes are appropriate.

The *HLA* region has been excluded from epigenetic analyses in many cases due to the technical difficulty of obtaining reliable data for highly polymorphic regions from microarrays. The set of probes that were confirmed to be reliable in our study can be applied in other studies on the DNA methylation status of Japanese subjects. Here, we focused on the epigenetics of NT1, but these methods are applicable and should be useful in future studies of the *HLA* region for any other disease.

We compared the methylation rates between the patients with NT1 and the controls based on the set of reliable probes identified in our previous study. As a result, nine CpG sites for CD4^+^ T cells and 19 sites for CD8^+^ T cells were found to be differentially methylated positions (DMPs), and they were particularly located in the *HLA* class II region. None of these DMPs showed a significant difference when the effect of the DQB1*06:02 allele was taken into account, suggesting the association of the allele; in particular, the hypomethylation of cg00699601 in both CD4^+^ T cells and CD8^+^ T cells, and cg11404906 in CD8^+^ T cells was shown to be specific for the DQB1*06:02 allele. This result is in agreement with that of a previous report showing the effect of an *HLA-DRB1* allele on DNA methylation and gene expression^45^. On the other hand, other DMPs showed nominally significant *P* values even after correction for the status of the DQB1*06:02 allele. This is also a notable result as it suggests the possibility that not only the *DQB1* allele, but also some other factors are related to the hypomethylation of these sites.

Our study revealed that some of the DMPs in patients with NT1 can be attributed to the presence of the DQB1*06:02 allele, while yet-unidentified factors related to hypomethylation may be involved for other DMPs. Although further studies are required to examine whether these findings are related to the pathogenicity of NT1, these results appear to be in agreement with the results of previous studies that suggest an association between genetic factors, with DQB1*06:02 in particular*,* and environmental factors.

Since CD4^+^ T cells and CD8^+^ T cells have been reported to be associated with the onset of NT1 through different mechanisms, we analyzed them separately. We found that while 30% of the CpG sites with a *P* value < 0.05 were in common between the CD4^+^ T cells and CD8^+^ T cells in the analysis without correction for the effect of DQB1*06:02, only 14% remained in common after correction for the effect of DQB1*06:02. This suggests that the gene expression status differed in CD4^+^ T cells and CD8^+^ T cells due to the difference in the DNA methylation pattern, supporting the findings of previous studies^26–28^.

While many studies have focused on the strong associations with the nucleotide sequences of *HLA* genes^6–11^, we showed that there were changes in DNA methylation in the *HLA* genes. As DNA methylation is known to regulate the expression levels of nearby genes, we performed RNA sequencing to analyze the expression levels in the same subjects to examine the functional relevance of the changes in the methylation status.

We first showed that the expression level of the *HLA-DQB1* gene was higher in the patients than in the controls. However, contrary to our expectation, the subsequent determination of the *HLA* types revealed that the expression level of HLA-DQB1*06:02, which is an allele known to be crucial for NT1, did not differ between the patients and controls. Since *HLA* genes are known to show allele-specific differences in their levels of expression, and the expression level of the DQB1*06:02 allele was distinctively higher than the expression levels of the other alleles, we assume that the higher expression level of *HLA-DQB1* in NT1 was due to the difference in the number of HLA-DQB1*06:02-positive samples between the patients and controls (42/84 alleles and 8/84 alleles were HLA-DQB1*06:02 among the patients and controls, respectively).

Studies have showed that almost all patients with NT1 carry HLA-DQB1*06:02 in many populations^6–11^, and that the allele is associated with autoreactive CD8^+^ T cells that attack orexin neurons^27^, suggesting that the protein transcribed from HLA-DQB1*06:02 plays an essential role in the onset of NT1. However, since the allele is a relatively common allele in general populations, and the expression level of the allele was similarly high in both the patients and controls, it appears that the onset of NT1 cannot be explained solely by the quantity of the transcribed protein of HLA-DQB1*06:02. In addition, we found that *HLA-DQB1* alleles that play a protective role against NT1, were expressed at a significantly low level; this finding is consistent with several studies that showed the relationship of a combination of *HLA-DQB1* alleles with susceptibility to NT1^8,10,46^. In the stratified comparison, we showed that in the lower expression group, which included susceptibility alleles for NT1, such as HLA-DQB1*03:01 and HLA-DQB1*03:02^8,10,46^, there was no difference in the expression levels between the patients and controls. In contrast, in the higher expression group, which included protective alleles against NT1, such as HLA-DQB1*06:01 and HLA-DQB1*05:01^8,10,46^, the expression levels were significantly lower in the patients than in the controls. These findings suggest that in NT1, the relative expression level of DQB1*06:02 is higher due to the lower expression of alleles other than DQB1*06:02 in the DQB1 gene and that the higher relative expression of DQB1*06:02 might be associate with the susceptibility of NT1. Although the absolute expression level of HLA-DQB1*06:02 did not differ between the patients and controls, the reduced expression level of the other alleles in the patients made the relative expression level of the HLA-DQB1*06:02 allele high. Although the presentation of disease-relevant self-peptides is thought to be essential for NT1, our study indicated that the interaction of HLA-DQB1*06:02 and other *HLA-DQB1* alleles, in terms of their gene expression, might also be a functional component of the development of NT1.

The associations of HLA-DPB1*05:01 and HLA-DPB1*04:02, after controlling for the effect of the HLA-DQB1*06:02 allele, have been reported in Japanese, Chinese, and European populations^8,10,46^. However, we found no difference in the expression levels of *HLA-DPB1* alleles. Therefore, the disease mechanism underlying the association of *HLA-DPB1* with narcolepsy remains to be elucidated. Additionally, as HLA-DQB1*06:02 shows complete linkage disequilibrium with HLA-DRB1*15:01 in the Japanese population, we compared the expression levels of the *HLA-DRB1* alleles. However, none of the alleles were expressed at different levels between the patients and controls, suggesting that the onset of NT1 might be associated not with *HLA-DRB1*, but with *HLA-DQB1,* despite the strong linkage between the two alleles.

This study has several limitations. Firstly, the analysis was performed using methylation levels measured by the array, and validation of the results using alternative measurement methods such as pyrosequencing or next generation sequencing has not been conducted. Analyzing the *HLA* region is challenging due to its high polymorphisms, but in the future, it would be beneficial to confirm the reproducibility of the results using other experimental approaches. Secondly, in this study, the direct measurement of the impact of SNPs in probe sequences was not performed. Based on the results of previous studies, we classified SNPs that could potentially affect the probe's affinity. However, experimental validation could potentially identify more reliable probes. Finally, the sample size is relatively small. Increasing the sample size may enhance the ability to detect associations between NT-related methylation sites and the expression of individual *HLA* alleles. Despite these limitations, in this study, we performed an extensive analysis of DNA methylation in the *HLA* region to identify novel DMPs in patients with NT1. In addition, our further analysis of *HLA* gene expression revealed the possibility that the low expression level of *DQB1* alleles other than DQB1*06:02 in patients with NT1 is related to the onset of the disease. As DNA methylation in the *HLA* region has not yet been well studied, our assessment of the microarray probes is expected to be useful for and applicable to other medical studies.

## Supplementary Information

Supplementary Information.

Supplementary Table 2.

Supplementary Table 3.

## Data Availability

The dataset(s) supporting the conclusions of this article has been deposited into the DDBJ website (https://ddbj.nig.ac.jp/) with accession number, PRJDB15980.
