# Supplementary material for: Narcolepsy type I-associated DNA methylation and gene expression changes in the human leukocyte antigen region
Source: Sci Rep. 2023 Jun 28;13:10464. doi: 10.1038/s41598-023-37511-4 (PMC10307834; doi:10.1038/s41598-023-37511-4)
Supplement: Supplementary file 1 — Supplementary Information. [file 41598_2023_37511_MOESM1_ESM.docx]

**Supplementary fig1. Examination of the reliability of probes (MAF ≥ 0.05)**

The examination results of the SNPs with a MAF ≥ 0.05 as the baseline. (A) Number of SNPs classified according to the distance from 3’ end of the probe. More than 90% of the probes after general filtering did not have any SNP in the influential region. (B)Number of probes that had one or more SNPs. Many of the SNPs were located in “ONSITE” or “5nt”. (C)Number of probes that had multiple SNPs within “ONSITE”, “5nt”, and “10nt” (blue) and “ONSITE” and “5nt” (red). These probes were theoretically estimated to have low reliability due to their high polymorphism. (D) Histogram of the number of probes that had one or more SNPs within “ONSITE”, “5nt”, and “10nt” in the *HLA* region. Most of them targeted CpG sites in the Class I and Class II region.

**Supplementary fig2. The expression levels of the *HLA-DPB1* alleles**

The expression levels of *HLA-DPB1* alleles in the patients and controls. The expression levels refer to the transcript abundances calculated from the read counts normalized by allele lengths with arcasHLA, and the total read number standardized to 1 million.

**Supplementary fig3. The expression levels of the *HLA-DRB1* alleles**

The expression levels of *HLA-DRB1* alleles in the patients and controls. The expression levels refer to the transcript abundances calculated from the read counts normalized by allele lengths with arcasHLA, and the total read number standardized to 1 million.

**Supplementary Table 1. The demographic characteristics of the samples**

**Supplementary Table 4. P values and log fold-change (logFC) of the comparison of the *HLA* gene expressions between the patients and the controls**
